# Frequency and Stratification of Epileptogenic Lesions in Elderly With New Onset Seizures

**DOI:** 10.3389/fneur.2018.00995

**Published:** 2018-11-30

**Authors:** Maher Arabi, Maya Dirani, Roula Hourani, Wassim Nasreddine, Jaafar Wazne, Samir Atweh, Heba Samara, Abdel Rahman Shatila, Ahmad Beydoun

**Affiliations:** ^1^Ibn Sina Hospital, Kuwait City, Kuwait; ^2^American University of Beirut Medical Center, Beirut, Lebanon; ^3^Rafik Hariri University Hospital, Beirut, Lebanon

**Keywords:** new onset epilepsy, MRI, Elderly, epiletogenic, epilepsies, partial

## Abstract

**Objective:** To evaluate prospectively the frequency of epileptogenic lesions in a consecutive cohort of elderly patients presenting with new onset unprovoked seizures, and who underwent a complete evaluation including dedicated epilepsy protocol MRI.

**Methods and materials:** We included all consecutive patients 60 years or older who participated in a prospective study on new onset epilepsy. The work-up included the acquisition of a dedicated epilepsy protocol MRI and a 3 h video/EEG recording. We evaluated the frequency and types of epileptogenic lesions in the whole cohort and stratified those variables by age, gender, types and number of seizures at presentation. We also correlated the EEG findings with the clinical characteristics and neuroimaging results.

**Results:** Of the 101 patients enrolled in the study and who underwent an epilepsy protocol MRI, an epileptogenic lesion was identified in 67% of cases. The most common etiologies were vascular events, followed by tumoral causes and traumatic brain injuries. Epileptogenic lesions were more likely to be identified in patients who presented with only focal aware and impaired awareness seizures. In addition, patients with tumoral epilepsy were significantly more likely to only experience those seizure types compared to patients with other pathological substrates. Interictal/ictal discharges were detected in the EEG of 21% of patients. Epileptiform discharges were significantly more frequent in patients with an epileptogenic lesion on brain MRI, especially in those with a brain tumor.

**Conclusions:** Our results stress the importance of obtaining a dedicated epilepsy protocol MRI in elderly patients with new onset seizures. An epileptogenic lesion will be identified in approximately two thirds of patients with important implications regarding initiation of treatment. In addition, the data underscore the value of distinguishing the types of seizures experienced at presentation as this will apprise the treating physician on the likelihood of identifying an epileptogenic lesion and on the probable etiologies.

## Introduction

Although population based studies established that the incidence of seizures increases markedly after 60 years of age(1–6), relatively few studies focusing on epilepsy in the elderly have been conducted. Seizures in the elderly can sometimes be difficult to ascertain and the etiology of seizures in this age group is different than that reported in younger age groups (7–9). The yield of detecting underlying lesions responsible for the seizures has increased over time, especially due to the more frequent use of brain MRI, known to have a substantially higher sensitivity compared to head CT scans (10). However, a number of studies have reported that 50% or more of elderly patients with new onset epilepsy have no detectable epileptogenic lesion on neuroimaging (11–17).

The purpose of this study is to evaluate prospectively evaluate the frequency of epileptogenic lesions in a consecutive cohort of elderly patients presenting with new onset unprovoked seizures, and who underwent a complete evaluation including a dedicated epilepsy protocol MRI and a 3 h video/EEG recording. The secondary objectives are to determine the types of epileptogenic lesions in this age group and to stratify the frequencies and types of epileptogenic lesions according to age, gender, types, and number of seizures at baseline. We also correlated the EEG findings with the clinical characteristics and neuroimaging results.

## Materials and methods

### Study design

Patients identified for this study were enrolled in an ongoing prospective study conducted on children and adults with new onset seizures. This is a centralized study conducted at the American University of Beirut Medical Center (AUBMC) in association with the Lebanese Chapter of the International League against Epilepsy (ILAE). Neurologists from across Lebanon are referring patients with newly diagnosed seizure/epilepsy to the AUBMC where a full medical history, including a detailed description of the spells is reviewed by two epileptologists and physical/neurological examinations are performed. As per protocol, the work-up on each patient includes a 3-h sleep deprived video-EEG and an epilepsy protocol brain MRI. Patients are subsequently evaluated by phone calls to inquire about adverse events (in case treatment was initiated) and have follow-up visits and repeat EEGs as clinically indicated.

### Patient characteristics

For the purpose of this study, we used the United Nations definition of elderly as patients aged 60 years or older (18) as was done in a number of previous studies (8, 19–21).

All patients 60 years or older, who experienced one or more unprovoked seizures between November 2010 and April 2015 were included in this study. Patients with seizure onset prior to the age of 60 years and those with acute symptomatic seizures (22) were excluded. We specifically reviewed the work-up performed at the time of the seizures, inquired about any recent intake or discontinuation of medication and about alcohol history to ascertain that the seizures were unprovoked (23).

The seizure types were classified in accordance with the most recent ILAE classification (24). Two or more seizures within 24 h were categorized as representing a single seizure. Generalized convulsive status epilepticus was defined as a continuous seizure lasting at least 5 min or two or more discrete seizures with an incomplete recovery of consciousness between seizures (25),whereas focal status epilepticus was defined as a single focal aware or impaired awareness seizure lasting more than 30 min or repeated seizures without full recovery of consciousness between seizures (26).

### Brain MRIs

Brain MRIs were obtained from a 1.5 or 3 Tesla scanner (Ingenia, Phillips) using an epilepsy imaging acquisition protocol that included 3D T1 (1 mm slice thickness) and 3D fast fluid-attenuated inversion recovery (FLAIR; 0.9 or 1 mm slice thickness) of the whole brain with multiplanar reconstruction, axial and coronal inversion recovery (2 mm slice thickness), axial T2 TSE and T2 FFE (4 mm slide thickness) and axial diffusion weighted images (4–5 mm slice thickness).

All MRIs were interpreted by a neuroradiologist with extensive experience in the evaluation of patients with epilepsy and who was blinded to the patient's history. MRIs were classified as normal, abnormal but non-epileptogenic, and epileptogenic based on previously published criteria (27, 28). For the purpose of this study, only lesions likely relevant to the cause of the seizure and concordant with the seizure semiology were considered epileptogenic. Epileptogenic lesions were subsequently categorized under vascular (stroke, intracerebral hemorrhage, etc.), traumatic (subdural hematoma, subarachnoid hemorrhage, intracerebral hematoma), tumoral (glioma, metastastasis), or others (vasculitis, post-radiation gliosis, etc). MRI abnormalities consisting of isolated subcortical high T2 signal, leukoaraiosis, brain atrophy (irrespective of the severity of atrophy), or post-operative encephalomalacia in the absence of cortical gliosis were not considered epileptogenic and the patients were labeled as having cryptogenic epilepsy. Leukoaraiosis was documented when isolated or confluent lesions of variable size with high T2 signal localized in the periventricular areas and/or deep white matter were seen (29).

### EEGs

All patients underwent a 3-h video/EEG recording. The studies were acquired with digital EEG systems (Natus^R^ Neurodiagnostics) using 21 electrodes according to the international 10–20 system and interpreted by a board certified epileptologist with more than 20 years of experience in EEG interpretation. Patients were routinely sleep deprived the day prior to the recording except when the studies were acquired on an emergency basis.

### Ethical approval

This study was approved by the Institutional Review Board of the AUBMC and all patients signed an informed consent form.

### Statistical analyses

We calculated the percentage of patients with an epileptogenic lesion on brain MRI and compared the frequency and types of epileptogenic lesions according to age, gender, and types and number of seizures at baseline. We also evaluated the types of EEG abnormalities and correlated them with a number of clinical and neuroimaging features. For continuous variables, descriptive statistics including mean, median, range, and frequencies with percentages were calculated. Statistical analysis was performed using Chi-square test or Fisher exact test for categorical variables. Significant *P*-values were set at <0.05.

## Results

### Demographic variables

Of the 117 consecutive patients enrolled in the study, 16 patients were excluded for the following reasons: two patients with a cardiac valve replacement could not undergo a brain MRI and were evaluated with a head CT, Nine additional patients were only evaluated with a head CT and refused to have a brain MRI, four underwent a non-epilepsy protocol MRI on a low Tesla machine and declined to undergo a repeat MRI and one patient refused to undergo any imaging studies at all.

Therefore, a total of 101 patients (males 57%, females 43%), with a mean and median age of 72 years (range: 60.5–86.5 years), were included in this study. The patients were evaluated within a median of 4 days after their seizure (range: 0–77 days). At the time of their initial evaluation, 56 patients presented with a single seizure while 45 patients experienced more than one seizure. 48 patients experienced focal to bilateral tonic-clonic seizures (40 presented with focal to bilateral tonic-clonic seizures only and 8 experienced in addition focal aware or impaired awareness seizures), whereas 53 patients only experienced focal aware and/or impaired awareness seizures. 18 patients presented with more than one seizure within a 24 h period (range 2–6) while 4 patients presented with status epilepticus (two patients with focal aware status epilepticus and two with focal impaired awareness status epilepticus were confirmed with video/EEG recordings). None of the patients included in this study previously experienced an acute symptomatic seizure.

### MRI findings

**Figure 1:**
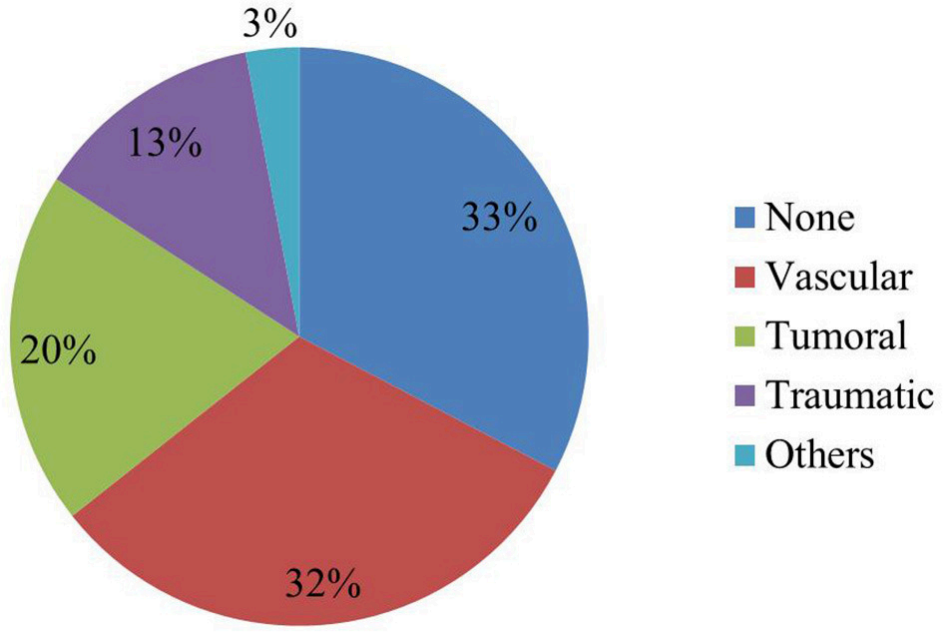
Frequencies of epileptogenic lesions stratified by etiological categories.

Overall, 68/101 elderly patients (67%) were found to have an epileptogenic lesion on brain MRI. The frequencies of epileptogenic lesions stratified by etiological categories are shown in Figure 1. The most common type of epileptogenic lesion was vascular, identified in 32 patients and accounted for 47% of cases in whom an epileptogenic lesion was identified. The vast majority (88%) had evidence of a prior ischemic infarction involving the cortex, 9% of patients were diagnosed with an amyloid angiopathy with several cortical microbleeds (evidenced by hemosiderin deposition on the T2 FFE sequence) and one patient had a remote and resolving spontaneous occipital hemorrhage (Table 1). The second most common type of epileptogenic lesion was tumoral, identified in 20 patients and accounting for 29% of cases with an epileptogenic lesion. Metastases were substantially more common than primary glioma in elderly patients who presented with new onset seizures (Table 1). Patients with multiple brain lesions and a primary tumor were assumed to have a metastasis. Those with a single lesion were diagnosed by pathology after resection or biopsy. Epileptogenic lesions associated with traumatic injuries were detected in 13 patients and accounted for 20% of cases with an epileptogenic lesion. The lesions most commonly consisted of cortical gliosis with and without associated encephalomalacia, chronic subdural hematoma and chronic traumatic intracerebral bleed (Table 1). Three patients, accounting for 4% of cases with an epileptogenic were found to have epileptogenic lesions caused by CNS lupus, post-radiation gliosis and multiple sclerosis with a cortical lesion involving the left inferior frontal gyrus.

**Table 1:** Subtypes of epileptogenic lesions stratified according to each category.

**Type of epileptogenic lesion**	**Subtype (%)**
Vascular (*n* = 32)	Ischemic cortical infarction (88%) Amyloid angiopathy (9%) Intracerebral hemorrhage (3%)
Tumoral (*n* = 20)	Metastasis (85%) Glioma (15%)
Traumatic (*n* = 13)	Gliosis ± encephalomalacia (54%) Chronic subdural hematoma^*^ (38%) Intracerebral hemorrhage^*^ (15%)
Others (*n* = 3)	Multiple sclerosis (33%) CNS lupus (33%) Post radiation gliosis (33%)

* *One patient had both a chronic subdural hematoma as well as a traumatic intracerebral hemorrhage*.

Four patients had a normal MRI and 29 patients had abnormal but non-epileptogenic MRIs. Leukoaraiosis was the most common abnormality (69%), followed by diffuse cortical atrophy (17%) and non-specific subcortical increased T2 signal.

Of the 15 patients excluded from the study and who underwent an imaging study, 6 (40%) were found to have an epileptogenic lesion (5 strokes and one primary brain tumor). The yield of detecting an epileptogenic lesion in patients who underwent a dedicated epilepsy protocol brain MRI was significantly higher compared to those who underwent a head CT or low tesla MRI imaging (*P* = 0.04).

### Stratification by age subgroups

No significant difference in mean age at time of index seizure was found between patients with an epileptogenic lesion on brain MRI and those with a cryptogenic etiology (Table 2). Although the presence of an epileptogenic lesion was highest in the 8th decade, followed by the 7th decade and lowest in the 9th decade, this difference did not reach statistical significance (Chi Square, *P* = 0.09) (Table 3). Vascular lesions were the most common epileptogenic lesion in all three decades, while the frequency of tumoral lesions progressively dropped across the three decades and traumatic lesions were more common in the 9th decade (Table 3).

**Table 2:** Demographic, clinical, EEG, and treatment variables stratified according to the presence or absence of an epileptogenic lesion on brain MRI.

	**MRI epileptogenic (*n* = 68)**	**MRI non-epileptogenic (*n* = 33)**	***P*-value**
Mean age at index seizure (years)	71.2	72.4	NS
**Gender**			
Male	41/58 (70.7%)	17/58 (29.3%)	NS
Female	27/43 (62.8%)	16/43 (37.2%)	
**Number of seizures (%)**			
single seizure multiple seizures	39/68 (57.4%) 29/68 (42.6%)	17/33 (51.5%)16/33 (48.5%)	NS
Seizure cluster or SE (%)	18/50 (36.0%)	4/29 (13.8%)	0.1
Seizure types (%)^*^			
Focal aware	23/68 (33.8%)	5/33 (15.2%)	0.015
Focal impaired awareness	28/68 (41.2%)	8/33 (24.2%)	
FBTC	26/68 (38.2%)	22/33 (66.7%)	
**Results of initial EEG**			
No IED/ictal discharges	50/68 (73.5%)	30/33 (90.9%)	0.04
IED/ictal discharges	18/68 (26.5%)	3/33 (9.1%)	
% started on AED	68/68 (100%)	33/33 (100%)	NS
**Initial AED**			
PHT	31/68 (45.6%)	12/33 (36.4%)	NS
LEV	13/68 (19.1%)	4/33 (12.1%)	
VPA	13/68 (19.1%)	8/33 (24.2%)	
CBZ	9/68 (13.2%)	5/33 (15.2%)	
LTG	2/68 (2.9%)	4/33 (12.1%)	

* *Some patients experienced more than one seizure type; FBTC, Focal to bilateral tonic-clonic seizures; PHT, Phenytoin; LEV, levetiracetam; VPA, valproate; CBZ, carbamazepine; LTG, lamotrigine; NS, not significant*.

**Table 3:** Frequencies and types of epileptogenic lesions stratified according to age.

**Age range**	**Epileptogenic lesion (%)**	**Type of epileptogenic lesion**
60–70 years (*n* = 46)	65.2%	Vascular (36.7%)Tumoral (36.7%)Traumatic (16.7%)Others (10.0%)
70–80 years (*n* = 40)	77.5%	Vascular (58.1%)Tumoral (25.8%)Traumatic (16.1%)
80–90 years (*n* = 15)	46.6%	Vascular (42.9%)Traumatic (42.9%)Tumoral (14.3%)

### Stratification by gender and number of seizures at presentation

Although epileptogenic lesions were less frequently identified in women (62.8%) compared to men (70.7%), this gender difference did not reach statistical significance (Table 2). Similarly, there was no significant gender difference in the types of epileptogenic lesions despite a higher frequency of traumatic lesions in men (24.4%) compared to women (11.1%). Likewise, no significant differences in the frequencies or types of epileptogenic lesions between patients who presented after a single or multiple seizures were found (Table 2).

### Stratification by presence or absence of seizure cluster or status epilepticus

Although patients who presented with a seizure cluster or status epilepticus were more likely to have an epileptogenic lesion on MRI, the difference did not reach statistical significance (*P* = 0.13, Fisher's exact test) (Table 2). In addition, the types of epileptogenic lesions did not significantly differ between those two groups.

### Stratification by seizure types

Patients with an epileptogenic lesion on brain MRI were more likely to experience focal aware or focal impaired consciousness seizures whereas those without epileptogenic lesions were more likely to experience focal to bilateral tonic-clonic seizures (Table 2).

Patients who presented with only focal aware and impaired awareness seizures were more likely to have an epileptogenic lesion (79.2%) compared to those who experienced a focal to bilateral tonic-clonic seizures (54.2%) (Chi-Square *P* = 0.007; Table 4). In addition, patients who presented with focal aware and impaired awareness seizures were more likely to have a brain tumor identified on brain MRI compared to patients who experienced a focal to bilateral tonic-clonic seizure (Fisher exact test, *P* = 0.002; Table 4).

**Table 4:** Frequencies and types of epileptogenic lesions stratified according to the types of seizures at presentation.

**Type of sz at presentation**	**Epileptogenic lesion (%)**	**Type of epileptogenic lesion**
Focal aware and impaired awareness seizures only (*n* = 53)	79.2%	Tumoral (42.9%)Vascular (38.1%)Traumatic (19.0%)
Focal to bilateral tonic-clonic seizures (*n* = 48)	54.2%	Vascular (61.5%)Traumatic (19.2%)Others (11.5%)Tumoral (7.7%)

Whereas, two thirds of patients with a cryptogenic brain MRI experienced a focal to bilateral tonic-clonic seizure, 90% of patients with a tumoral epilepsy only experienced focal aware and impaired awareness seizures at initial presentation (Table 5). Patients with vascular epileptogenic lesions were equally as likely to experience focal to bilateral tonic-clonic seizures or focal aware and impaired awareness seizures only at initial presentation (Table 5).

**Table 5:** Frequencies of seizure types at initial presentation stratified according to types of epileptogenic lesions.

**Type of epileptogenic lesion**	**Focal aware and impaired awareness seizures only**	**Focal to bilateral tonic-clonic seizures**
Cryptogenic (*n* = 33)	11(33.3%)	22 (66.7%)
Vascular (*n* = 32)	16 (50%)	16 (50%)
Tumoral (*n* = 20)	18 (90%)	2 (10%)
Traumatic (*n* = 13)	8 (61.5%)	5 (38.5%)

### EEG findings

The EEG was abnormal in 87 patients (86.1%) and normal in 14 (13.9%). The most severe abnormalities consisted of slowing (focal in 58 and generalized in 8), focal interictal discharges in 18 (17.8%), and ictal discharges in 6 (3 of whom also had interictal discharges). Therefore, a total of 21 patients (20.8%) had either interictal or ictal discharges recorded on their initial video/EEG study.

### EEG yield and clinical features

Although not statistically significant, the frequency of interictal/ictal discharges was highest in the 70–80 years age group (30.0%), followed by the 60–70 years age group (17.4%), with the lowest frequency in the patients older than 80 years (6.7%).

The yield of detecting interictal/ictal discharges on the EEG was not significantly impacted by the seizure type with epileptiform discharges recorded in 7/48 patients (14.6%) who experienced a focal to bilateral tonic-clonic seizures compared to 14/53 (26.4%) who only experienced focal aware and impaired awareness seizures at the time of their evaluation (*P* = 0.14).

Patients who presented with more than one seizure were more likely to show interictal/ictal discharges on the EEG (13/45, 28.9%) compared to those who presented with a single seizure (8/56, 14.3%). This statistical trend, however, failed to reach significance (*P* = 0.07).

### Patients who presented with status epilepticus or with recorded seizures on initial video/EEG

In the initial 3 h video/EEG recording, 6 patients (5.9%) had seizures recorded. Four of those were in clinical status as described above with seizures recorded on the EEG in two additional patients (one experienced a focal impaired awareness seizure and an electrographic seizure was recorded in the other). Five of those (86.3%), including all four patients with status epilepticus had epileptogenic lesions on brain MRI consisting of brain tumors in three (two metastases and one leptomeningeal carcinomatosis) and posttraumatic injuries in two (chronic subdural hematoma in one and posttraumatic gliosis in the second).

### Comparison of MRI and video/EEG findings between patients evaluated as inpatients and outpatients

Forty patients were evaluated in the emergency room at AUBMC and underwent a brain MRI and a 3 h video/EEG within 48 h of their seizure. The remaining 61 patients were evaluated as outpatients. The yield of detecting epileptogenic lesions on brain MRI was not significantly different between the two groups with epileptogenic lesions detected in 30/40 (75%) of inpatients compared to 38/61 (62.3%) of outpatients. Similarly, no significant correlation was found between the time of video/EEG recording from last seizure and the presence of interictal/ictal discharges on the EEG. When the EEG was performed within 48 h of the last seizure, interictal/ictal discharges were recorded in 11/42 patients (26.2%) compared to 10/59 (16.9%) when the EEG was performed more than 48 h later. This difference, however, did not reach statistical significance.

### Interictal/ictal discharges and MRI findings

There was a significant difference between the presence of interictal/ictal discharges and the presence of an epileptogenic lesion on brain MRI with epileptiform discharges recorded in 26.5% of patients with an epileptogenic lesion compared to 9.1% of those without (*P* = 0.04) (Table 2). Within the group of patients with epileptogenic lesions on MRI, the pathological substrate most frequently associated with epileptiform discharges on the EEG was the tumoral group (40.0%), followed by the traumatic group (30.8%), and the vascular group (15.6%). This difference however did not reach statistical significance.

In all patients, the seizure semiology was either concordant or at least not discordant with the location or lateralization of the epileptogenic lesion, when present. Of the 21 patients with interictal/ictal discharges on their video/EEG recording, 18 had an epileptogenic lesion on brain MRI. In 11 of those cases, the epileptiform discharges originated either from the same lobe (11 patients) or ipsilateral to the side of the lesion (7 patients).

### AED treatment

All patients included in this study were initiated on AED treatment. Phenytoin was the most common initial AED, administered to 42.6% of patients, followed by valproate (20.8%), levetiracetam (16.8%), carbamazepine (13.9%), and lamotrigine (5.9%) (Table 2). The long term follow-up of those patients and response to treatment will be the subject of a separate study.

## Discussion

This is the first prospective study that evaluated consecutive elderly patients with unprovoked new onset seizure or new onset epilepsy with a dedicated epilepsy protocol brain MRI. Our results indicate that 67% of those patients have a definite epileptogenic lesion. In addition, we documented that the most common etiologies were vascular events, predominantly ischemic cerebral infarctions, followed by tumoral causes and traumatic brain injuries.

Identifying an epileptogenic lesion is valuable in elderly patients with a single unprovoked seizure since it might influence the decision to initiate treatment and could lead to the diagnosis of epilepsy depending on the likehood of seizure recurrence (30). A recent study conducted in elderly patients with a single unprovoked seizure found that the presence of an epileptogenic lesion was the only variable that predicted seizure recurrence (9). The authors therefore recommended that treatment decisions in older patients presenting with a first seizure be based on established risk factors and not on age (9).

Our yield of detecting epileptogenic lesions is substantially higher than previous studies that reported neuroimaging abnormalities in 44–56% of elderly patients with epilepsy (9, 14, 16, 21) (Table 6). Those studies suffer from methodological flaws including retrospective designs (14, 16), neuroimaging evaluation consisting of a mixture of head CTs and non-epilepsy protocol brain MRIs (9, 16, 21) and a primary focus on the clinical characteristics and prognosis (9, 14, 16) (Table 6). Furthermore, some of those studies included various degrees of cerebral atrophy in the percentages of neuroimaging abnormalities (16) while others included patients with acute symptomatic seizures (21) (Table 6). For instance, a study that reported an etiological diagnosis in 47% of elderly patients with new onset seizures included 7% diagnosed with dementia and failed to specify the neuroimaging abnormalities detected on MRI (16). Another study reported that 48.8 and 55.8% of elderly patients with new onset seizures had evidence of a focal lesion on head CT and brain MRI, respectively (21). A substantial proportion of those patients (42%), however experienced acute symptomatic seizures due to a stroke, traumatic brain injury or metabolic abnormalities (21). The value of performing a dedicated epilepsy protocol brain MRI was shown in our study by demonstrating a significantly higher yield of detecting epileptogenic lesions (67%) compared to the excluded cohort that underwent head CT scan or low tesla brain MRI (40%).

**Table 6:** Previous studies reporting neuroimaging findings in elderly patients with epilepsy.

**Study**	***N***	**CT/MRI**	**% epileptogenic lesions**	**Types of epileptogenic lesions**	**Comments**
(21)	43	MRI (100%)	56%	Vascular: 30%Tumoral: 12%Traumatic: 0%Other: 4%	Patients with acute symptomatic seizures included
(15)	NR	MRI (100%)	33%	Vascular: NRTumoral: NRTraumatic: NROther: NR	Outpatient clinics
(14)	122	% MRI (Not reported)	45%	Vascular: 23%Tumoral: 9%Traumatic: 3%Other: 10%	Focused on clinical characteristics
(9)	139	% MRI (Not reported)	48%	Vascular: 32%Tumoral: 9%Traumatic: 4%Other: 3%	First seizure
(16)	70	% MRI (Not reported)	47%	Vascular: 17%Tumoral: 5%Traumatic: 2%Dementia: 7%Inflammation: 9%Others: 6%	% epileptogenic included 7% with dementia and 6% with metabolic disorders
(17)	72	% MRI (Not reported)	52%	Vascular: 15%Tumoral: 19%Traumatic: 4%Genetic: 8%Degenerative: 6%	Cohort of patients age 60 years or older at last visit
Our study	101	Dedicated epilepsy protocol MRI (100%)	67%	Vascular: 32%Tumoral: 20%Traumatic: 13%Other: 3%	Prospective trial

A recent prospective study evaluated patients from all age groups who presented to an outpatient first seizure clinic with a 1.5 or 3T brain MRI (15). Epileptogenic lesions were identified in 23% of the whole cohort and in 33% of patients 65 years and older (15). The substantially lower frequency of epileptogenic lesions in that study compared to ours is likely related to the fact that they only evaluated outpatients who presented to their clinic after a median of 24 days from their index seizure whereas our cohort consisted of inpatients as well as patients evaluated in the emergency rooms and outpatient clinics within a median of 4 days from their seizure.

In our study, only lesions known to be associated with seizures and concordant with the clinical semiology were considered epileptogenic (28). For instance, evidence of only small vessel disease (leukoaraiosis) on brain MRI was not categorized as epileptogenic and was identified in 69% of our patients considered to have a cryptogenic epilepsy. Some have suggested that leukoaraiosis is pro-epileptogenic due to its increased prevalence in elderly patients with seizures (31–33) and hypothesized the seizures to be due to blood flow diminution (34) or to occult cortical microinfarcts that escape detection with 3 Tesla brain MRI (33, 35). Similarly, although dementia is estimated to account for 10–20% of epilepsies in the elderly (36, 37), cortical atrophy which was found in 17% of our cohort was not considered epileptogenic.

We found that cerebrovascular disease, identified in 32% of our patients was the most common etiology of new onset seizures in the elderly. This result is concordant with previous studies that reported that cerebrovascular disease accounted for 34–50% of cases of epilepsy in the elderly (13, 14, 38). The second most identified epileptogenic lesion was tumoral, which was detected in 20% of patients, a result also in line with published data indicating that tumoral epilepsy accounts for 10–30% of seizures in the elderly (14, 39, 40). Epileptogenic lesions associated with traumatic injuries were identified in 13% of elderly patients, a result concordant with the percentages reported in the literature (41).

Fifty-Two Percent of patients in our cohort only experienced focal aware and impaired awareness seizures while the remaining 48% experienced focal to bilateral tonic-clonic seizures. Those results are consistent with previous studies that reported that 48–53% of elderly patients with new onset seizures present with focal aware and impaired awareness seizures (14, 16). An interesting and previously unreported finding is that patients who only experienced focal aware and impaired awareness seizures at presentation are significantly more likely to have an epileptogenic lesion identified on neuroimaging (79%) compared to those who experienced focal to bilateral tonic-clonic seizures (54%). The other previously unreported finding is that patients with tumoral epilepsy are significantly more likely to experience only focal aware and impaired awareness seizures compared to patients with other pathological substrates.

The mean annual incidence of SE in the elderly ranges between 15.5/100,000 and 25.9/100,000 (42), with the most common cause being acute symptomatic seizures related to vascular events or brain hypoxia (42). We found that 4% of elderly patients with new onset seizures presented in status epilepticus, with an epileptogenic lesion detected in all of those patients. Our results are consistent with those of a study that evaluated a cohort of elderly patients who presented in status epilepticus, with epileptogenic lesions detected in the vast majority and only 16% having a cryptogenic etiology (43).

The yield of detecting epileptiform discharges is known to be lower in the elderly population with epilepsy compared to younger age groups. In a study of 308 patients with epilepsy of various age groups, 56% of all patients had interictal epileptiform discharges detected on their first EEG (44). The frequency in the first four decades of life was 77, 60, 56, and 51% respectively. In the 51 patients aged 40 years or older, the frequency was 39% but this latter group was not further divided (44). Studies that evaluated the diagnostic EEG yield in patients with onset of epilepsy after the age of 60 years reported epileptiform discharges in 28% (45) and 29% of patients (46), frequencies comparable to the 21% yield found in our study. Although the cause of the lower yield of EEG in the elderly population is not completely elucidated, it is possible that the decreased number and complexity of synaptic connections with age may contribute to less synchronized neuronal discharges and subsequently less frequent epileptiform discharges detected on scalp EEG.

We found that patients with an epileptogenic lesion identified on brain MRI were significantly more likely to have epileptiform discharges recorded on their EEG. Although the vascular etiology was the most common in our cohort of patients with identified epileptogenic lesions on brain MRI, the tumoral etiology was the most commonly identified pathological substrate in patients with epileptiform discharges on EEG.

All of the patients included in this study were initiated on AED treatment. The most commonly used initial AEDs were phenytoin and valproate. This result reflects the fact that at the time this study was performed, these were the only two drugs with an available parenteral formulation in Lebanon. Recently, the parenteral formulation of levetiracetam and lacosamide were approved by our regulatory agencies, a fact that might impact on the choice of initial AED in this patient population. Of the newer generation AEDs, levetiracetam, and lamotrigine were the most frequently administered drugs. The response to treatment of the initial AED, long term follow-up and frequency of drug resistant epilepsy in our cohort will be the subject of a separate study.

The strength of our study is its prospective design, assessing a cohort of consecutive elderly patients with new onset unprovoked seizures evaluated by an epilepsy protocol brain MRI interpreted by a neuroradiologist with expertise in epilepsy and blinded to the clinical history.

Our results stress the importance of obtaining a dedicated epilepsy protocol MRI in elderly patients with new onset seizures. An epileptogenic lesion will be identified in approximately two thirds of patients, with important implications regarding initiation of treatment. In addition, they underscore the value of distinguishing the types of seizures experienced at presentation as this will apprise the treating physician on the likelihood of identifying an epileptogenic lesion on brain MRI and on the probable pathological substrates.

## Author contributions

MA, MD, RH, and AB contributed conception and design of the study. WN organized the database. WN, JW, SA, HS, AS and AB wrote sections of the manuscript. All authors contributed to manuscript revision, read and approved the submitted version.

### Conflict of interest statement

The authors declare that the research was conducted in the absence of any commercial or financial relationships that could be construed as a potential conflict of interest.
